# Mechanocatalytic Depolymerization of Cellulose With Perfluorinated Sulfonic Acid Ionomers

**DOI:** 10.3389/fchem.2018.00074

**Published:** 2018-03-22

**Authors:** Ayman Karam, Prince N. Amaniampong, José M. García Fernández, Claudio Oldani, Sinisa Marinkovic, Boris Estrine, Karine De Oliveira Vigier, François Jérôme

**Affiliations:** ^1^INCREASE (FR Centre National De La Recherche Scientifique 3707), ENSIP, Poitiers, France; ^2^Institut de Chimie des Milieux et Matériaux de Poitiers, Université de Poitiers, Centre National de la Recherche Scientifique, ENSIP, Poitiers, France; ^3^Instituto de Investigaciones Químicas, CSIC—University of Sevilla, Sevilla, Spain; ^4^Solvay Speciality Polymers, Bollate, Italy; ^5^ARD-Agro-Industrie Recherches et Développements, Green Chemistry Department, Route de Bazancourt, Pomacle, France

**Keywords:** cellulose, depolymerization, mechanocatalysis, Aquivion, biomass

## Abstract

Here, we investigated that the mechanocatalytic depolymerization of cellulose in the presence of Aquivion, a sulfonated perfluorinated ionomer. Under optimized conditions, yields of water soluble sugars of 90–97% were obtained using Aquivion PW98 and PW66, respectively, as a solid acid catalyst. The detailed characterization of the water soluble fraction revealed (i) the selective formation of oligosaccharides with a DP up to 11 and (ii) that depolymerization and reversion reactions concomitantly occurred during the mechanocatalytic process, although the first largely predominated. More importantly, we discussed on the critical role of water contained in Aquivion and cellulose on the efficiency of the mechanocatalytic process.

## Introduction

With the transition of our society to a more sustainable development, the manufacture of chemicals, and fuels from renewable feedstocks has become a priority (Huber et al., 2006; Corma et al., 2007; Dhepe and Fukuoka, 2008; Rinaldi and Schüth, 2009a,b; Bozell and Petersen, 2010; Climent et al., 2011; Van de Vyver et al., 2011; Zhou et al., 2011; Gallezot, 2012; Luterbacher et al., 2014; Yabushita et al., 2014; Wang et al., 2015). In this context, due to its low cost, large availability and non-edibility, cellulose, a biopolymer made of β-1,4 linked D-glucose units, represents an attractive raw material. However, cellulose is highly recalcitrant to chemical processing due to the existence of a robust hydrogen bond network, both intrachain between glucose monomers in a single polymer strand and interchain between adjacent polymer chains, van der Waals interaction and electronic effects (Klemm et al., 2005; Shen and Gnanakaran, 2009; Siró and Plackett, 2010; Moon et al., 2011), which protect the glycosidic bond against hydrolysis, a pre-requisite step for the conversion of cellulose to soluble products. Hence, in many cases, the hydrolysis of cellulose requires high temperature or pressure, which leads to the concomitant formation of unwanted byproducts and thus to tedious work-up procedures.

Recently, mechanocatalytic depolymerization of lignocellulosic biomass has emerged as a contemporary frontier in biorefining (Zakrzewska et al., 2010; Groote et al., 2013; Zhang and Jérôme, 2013; Käldström et al., 2014; Kaufman Rechulski et al., 2015). This technology utterly overcomes the challenges and drawbacks posed by the recalcitrance of lignocellulose, in particular thanks to a synergistic effect between mechanical forces and catalysis (Beyer and Clausen-Schaumann, 2005; Barraud et al., 2008; Carrasquillo-Flores et al., 2013). Although the use of milling for altering the behavior of cellulose has been an old-age practice, as well as the influence of mechanical grinding on the reactivity of cellulose, the mechanocatalytic depolymerization of cellulose has garnered momentum in recent years (Hick et al., 2010; Meine et al., 2012). Mechanical forces provide energy to alter the crystalline structure and to reduce the particle size of cellulose. More importantly, it also changes the conformation of cellulosic chains and that of the glucose units, thereby lowering the *exo*-anomeric effect responsible to a large extent for the high stability of the β-1,4 glycosidic bond in cellulose (Hick et al., 2010; Carrasquillo-Flores et al., 2013; Loerbroks et al., 2013; Schmidt et al., 2016). Hence, by combining mechanical forces and an acid catalyst, cellulose was depolymerized in a large extent to water-soluble products that can be further processed into other valuable platform chemicals (Hick et al., 2010; Käldström et al., 2014; Schüth et al., 2014; Kaufman Rechulski et al., 2015). In comparison to the classical depolymerization of cellulose in acidic water, the activation energy barrier associated to the mechanocatalytic depolymerization was reduced by 66%, highlighting the synergistic effect between mechanical forces and catalysis (Kaufman Rechulski et al., 2015). In addition, in contrast to the classical ball-milling of cellulose, the overall energy required for the mechanocatalytic depolymerization of cellulose is much lower thanks to much shorter reaction times (2–6 h, depending on the milling mode, vs. 24–48 h for classical ball-milling).

**GRAPHICAL ABSTRACT:**
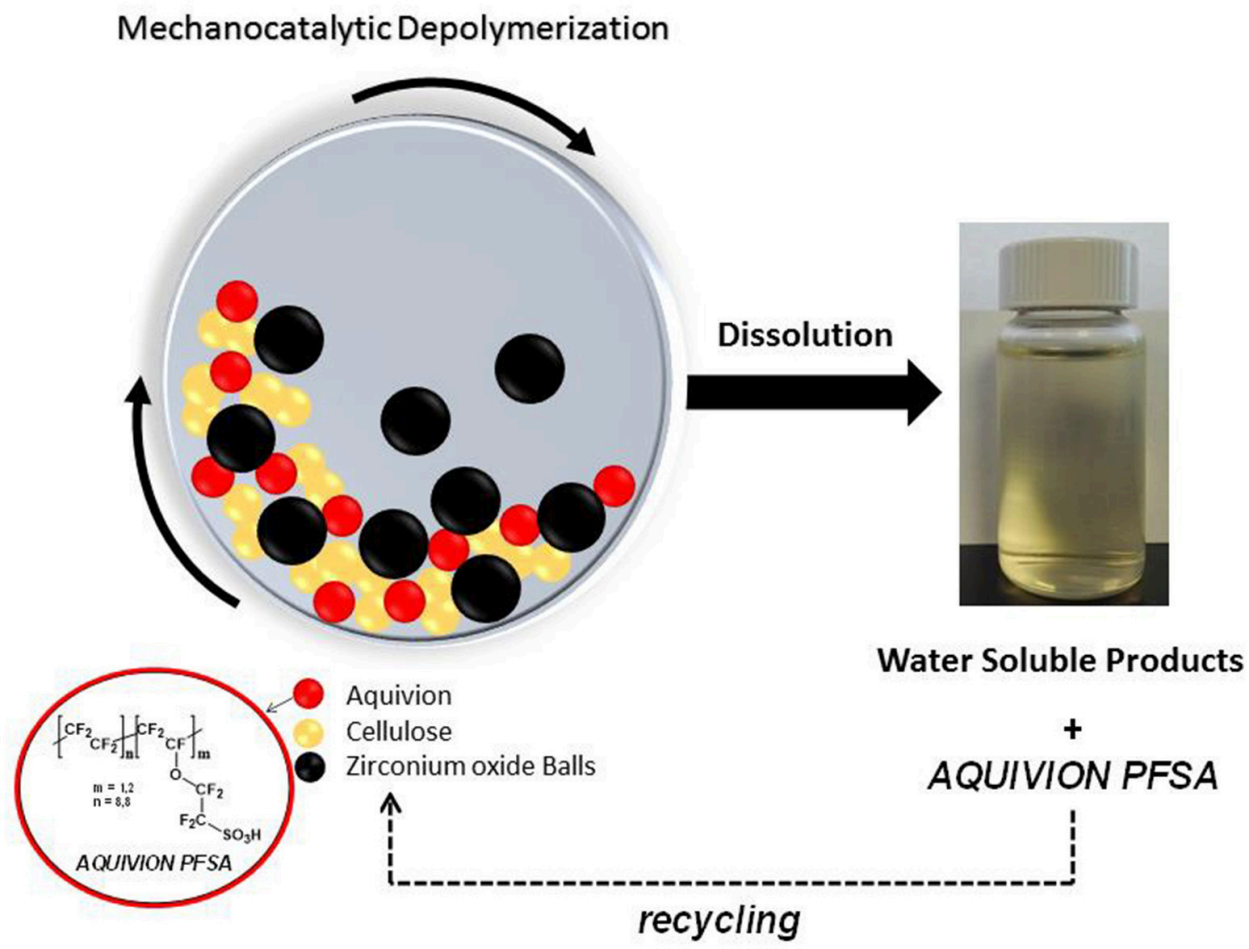
Aquivion PFSA, a sulfonated perfluorinated ionomer, is capable of depolymerizing selectively microcrystalline cellulose to oligosaccharide (DP up to 11).

For an effective mechanocatalytic depolymerization of cellulose, the targeted catalyst must be mechanically robust, and possesses sites that are physically accessible and chemically active. Planetary mills, shaker mills, attrition mills, and rolling mills are few examples of mills that promote an intimate contact between catalysts and cellulose during mechanocatalysis processes (Schell and Harwood, 1994; Suryanarayana, 2001; Esteban and Carrasco, 2006; Bitra et al., 2009). Recently, Rinaldi and Schüth reported the mechanocatalytic depolymerization of cellulose in the presence of about 10 wt% of sulfuric acid (Schüth et al., 2014). Remarkably, about 90% of cellulose was converted to a water-soluble fraction, which is composed of low molecular weight oligosaccharides with a degree of polymerization in a window 1–7. One drawback associated to this pathway is the removal of sulfuric acid at the end of the mechanocatalytic process, which is a tedious step unless the resulting low molecular weight oligosaccharides are further processed through an acid-catalyzed reaction. Blair and co-workers reported that water-soluble products can be obtained in good yields (~70–80%) upon ball milling of cellulose in the presence of inorganic solid acids, particularly dealuminated kaolinite (Hick et al., 2010). It was observed that kaolinite caused a rapid depolymerization of cellulose thanks to its exfoliation during the milling. However, the selectivity to low molecular weight oligosaccharides was lower than in the case of H_2_SO_4_ due to the side formation of levoglucosane and brown colored chemicals, presumably humins or furanic derivatives (Hick et al., 2010; Shrotri et al., 2013).

Recently, we reported that cellulose can be depolymerized to low molecular weight oligosaccharides by milling cellulose with Aquivion PW98, a strongly acidic perfluorinated sulfonic acid ionomer (H_0_ = −12, similar to H_2_SO_4_) (Karam et al., 2017). It was shown that Aquivion PW98 was chemically and mechanically resistant to the milling, permitting its long term recycling without altering its performances. In this article, we investigate the effect of the proton loading, stirring rate and Aquivion/cellulose mass ratio on the mechanocatalytic process. In particular, we point out the critical effect of water, even in trace amount, on the depolymerization rate of cellulose.

## Materials and methods

### Reagents

Microcrystalline cellulose (Avicel PH200, FMC Biopolymer) was utilized to investigate the performance of different solid catalysts.

### Catalyst synthesis and mechanocatalytic depolymerization of cellulose

#### Catalyst synthesis

SBA-15-SO_3_H catalyst was prepared following a reported procedure (Karam et al., 2007). In a typical synthesis process, pluronic (4 g) was dissolved in 125 g of aqueous HCl (1.9 M) and stirred at room temperature. The solution was then heated at 40°C before addition of 7.7 g (0.0369 mol) of TEOS. After stirring for 45 mn, MPTMS (0.8 g, 0.0041 mol) and 0.0369 mol of 35% H_2_O_2_ was added. The solution was then stirred for 24 h at 40°C and aged into a teflon autoclave for an additional 24 h at 100°C. The resulting solid was finally collected by filtration and thoroughly washed with water. The recovered SBA-15-SO_3_H was dried in an oven at 50°C for 18 h.

CMK-3-SO_3_H was synthesized *via* a reported procedure by Jun et al. (2000). Typically, the calcined SBA-15 was impregnated with aqueous solution of sucrose containing sulfuric acid, 1 g of SBA-15 was added to a solution obtained by dissolving 1.25 g of sucrose and 0.14 g of H2SO4 in 5 g of H2O. The mixture was placed in a drying oven for 6 h at 373 K, and subsequently the oven temperature was increased to 433 K and maintained there for 6 h. The sample turned dark brown or black during the treatment in the oven. The silica sample, containing partially polymerized and carbonized sucrose at the present step, was treated again at 373 and 433 K using the same drying oven after the addition of 0.8 g of sucrose, 0.09 g of H2SO4, and 5 g of H2O. The carbonization was completed by pyrolysis with heating to typically 1173 K under vacuum. The carbon—silica composite obtained after pyrolysis was washed with 1 M NaOH solution (50 vol % ethanol−50 vol % H2O) twice at 373 K or 5 wt % hydrofluoric acid at room temperature, to remove the silica template. The template-free carbon product thus obtained was filtered, washed with ethanol, and dried at 393 K. Thereafter, the recovered mesoporous carbon (so-called CMK-3) was suspended in concentrated H_2_SO_4_ (1 g of solid per 20 mL of acid) and stirred overnight. The CMK-3-SO_3_H was washed several times with distilled water and then dried in an oven at 60°C overnight.

Aquivion PW66, PW79, PW87, and PW98 were used without further pretreatment as received from Solvay Specialty Polymers.

#### Mechanocatalytic depolymerization of cellulsoe

Various amounts of cellulose and catalyst were ground using a planetary ball-mill (Retsch MP100). The mixture of catalyst and cellulose were ground in a 125 mL bowl made of Zirconium Oxide, utilizing 20 of 10 mm balls made of the same material as the milling bowl. The experiments were performed at desired rate for a desired time as described in the main manuscript for each conditions investigated.

### Determination of solubility

After each milling, the milled mixture of cellulose and catalyst was recovered. The determination of solubility involved three parts, dispersion, filtration, and drying. The dispersion is carried out in a 20 mL flacon, weighing 300 mg of the solid mixture and 20 mL distilled water, stirring and leaving it in an ultrasonic bath for 2 h. The mixture is filtered through a 47 mm Millipore Pyrex Filter Holder; the PFTE filter has a pore size of 0.22 μm. The filter containing the solid after filtration is placed in a petri dish in the oven at 60°C overnight. The final mass is measured by the difference between the filter containing the dry solid at ambient temperature and the initial mass of the filter.

## Results and discussions

**SCHEME 1:**
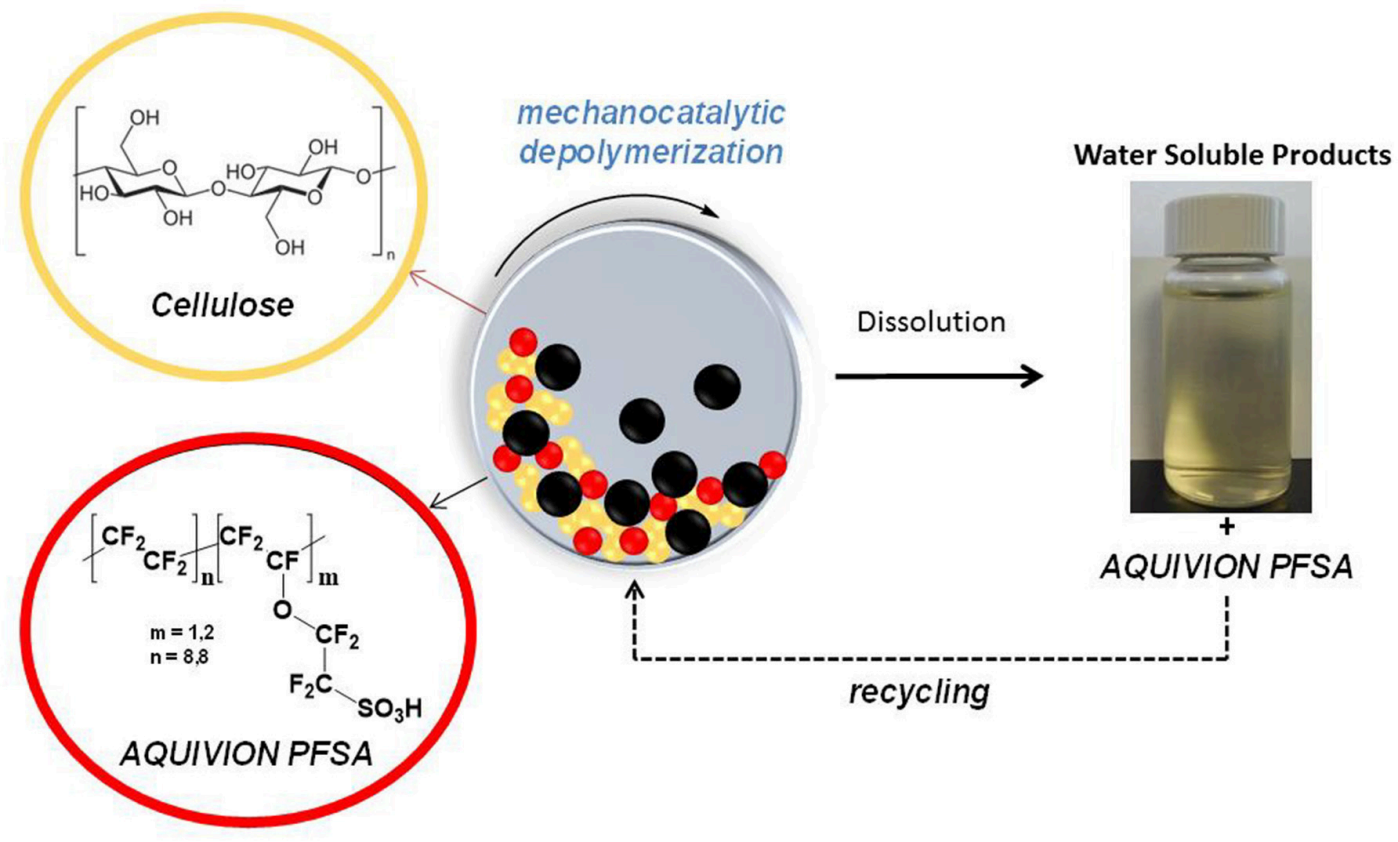
Mechanocatalytic depolymerization of cellulose to water soluble oligomers.

Aquivion PW98, sulfonated SBA-15 and sulfonated mesoporous carbon (CMK-3-SO_3_H) were either synthesized, or purchased, and initially screened as potential solid acid catalysts for the effective depolymerization of cellulose *via* ball-milling. More information on these solid acid catalysts are provided in the supporting information (SI). For starting experiments, 1 g of cellulose was mixed with 0.5 g of solid acid catalyst and stirred at 400 rpm in a planetary ball-mill for 24 h. The influence of experimental parameters such as the cellulose/catalyst mass ratio, stirring rate or reaction time is discussed later. The mechanocatalytic process is described in detail in the supporting information (section Introduction), and a schematic representation is shown in Scheme 1.

A < 5% solubility was observed when microcrystalline cellulose (MCC) was ball-milled for 24 h without any catalyst (Table 1, entry 1), in line with literature reported investigations (Meine et al., 2012). Treatment of neat MCC in a planetary ball mill in the presence of different solid acid catalysts remarkably improved the dissolution of the resulting product in water, showing the significance of intrinsic acid properties of solid acid catalysts in cleaving the β-1,4 glycosidic bonds in cellulose structures during the milling (Table 1). More information on the structure of products formed are provided at the end of the article.

**Table 1:** Mechanocatalytic depolymerization of cellulose in the presence of different solid acid catalysts^a^.

**Entry**	**Catalyst**	**H^+^ exchange capacity (mmol/g)**	**Solubility (%)^b^**
1	Blank	–	< 5
2	Aquivion PW98	1.0	90
3	SBA-SO_3_H	0.2	60
4	CMK-3-SO_3_H	0.7	87
5	Kaolinite (KGa-2)	–	50
6	Aquivion PW66	1.45	99
7	Aquivion PW79	1.26	32
8	Aquivion PW87	1.15	80

a Reaction conditions: Mass of Cellulose, 1 (g); Mass Catalyst, 0.5 (g); 400 rpm, Ball-milling time, 24 h; 20 Zirconium Oxide-balls d_MB_ = 10 mm;

b *± 8%*.

Aquivion PW98 catalyst led to the highest MCC solubility in water (~80%) followed by CMK-3-SO_3_H (87%) and SBA-SO_3_H (60%) (Table 1, entries 2–4). In our planetary ball-mill, kaolinite was found the least performant catalyst, leading to a product with a water solubility of only 50% (Table 1, entry 5). A significant difference of performance between the different tested Aquivion catalysts was observed (Table 1, entries 2, 6–8). The best result was obtained with Aquivion PW66 (~99 %) followed by PW98 (90%), PW87 (80%), and PW 79 (32%). Interestingly, there is no correlation between the proton loading of Aquivion samples and their efficiencies in the mechanocatalytic depolymerization of cellulose (Table 1, entries 2, 6–8). This result prompted us to investigate the humidity content of all the Aquivion catalysts used in this investigation, which will be discussed later in this manuscript.

**Figure 1:**
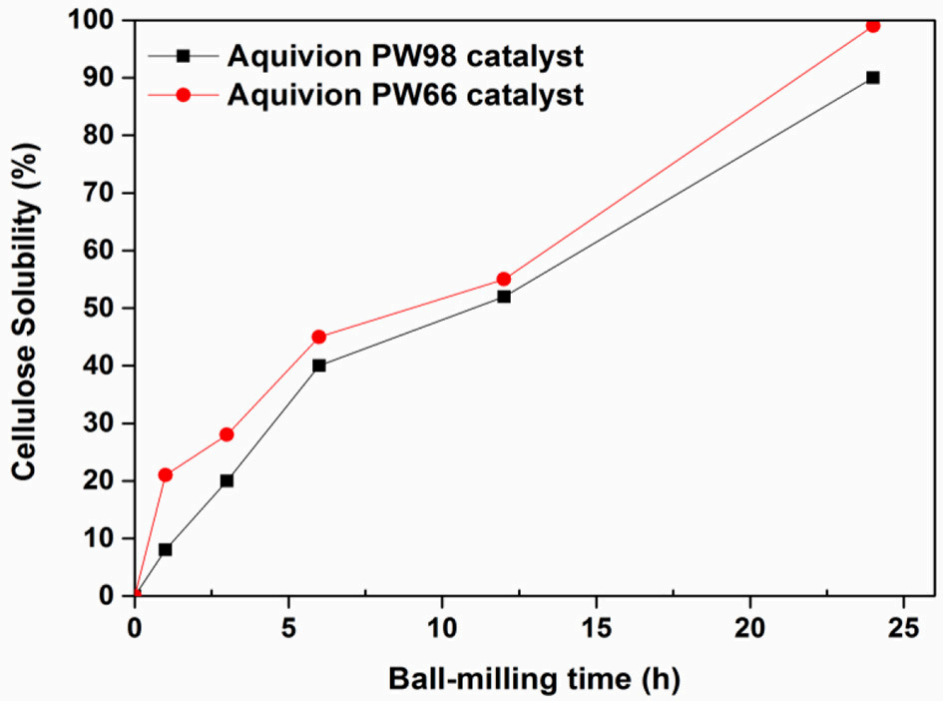
Kinetic profile of the mechanocatalytic depolymerization of cellulose with Aquivion PW 98 and PW66 (mass of cellulose, 1 g; catalyst, 0.5 g; milling speed, 400 rpm; zirconium oxide balls diameter, d_MB_, 10 mm).

Next, the effect of ball-milling time over the most active solid catalysts (Aquivion PW66 and PW98) identified in our preliminary investigations on the production of water soluble products from MCC was investigated. As shown in Figure 1, the solubility of ball-milled MCC increased markedly with ball-milling time, reaching a maximum of 90 and 99% within 24 h with Aquivion PW98 and PW66, respectively. Although both Aquivion samples have a different proton loading (Table 1), Aquivion PW66 and PW98 exhibited similar kinetic profiles, suggesting that the by—SO_3_H groups of Aquivion does not directly govern the kinetic of the reaction.

**Figure 2:**
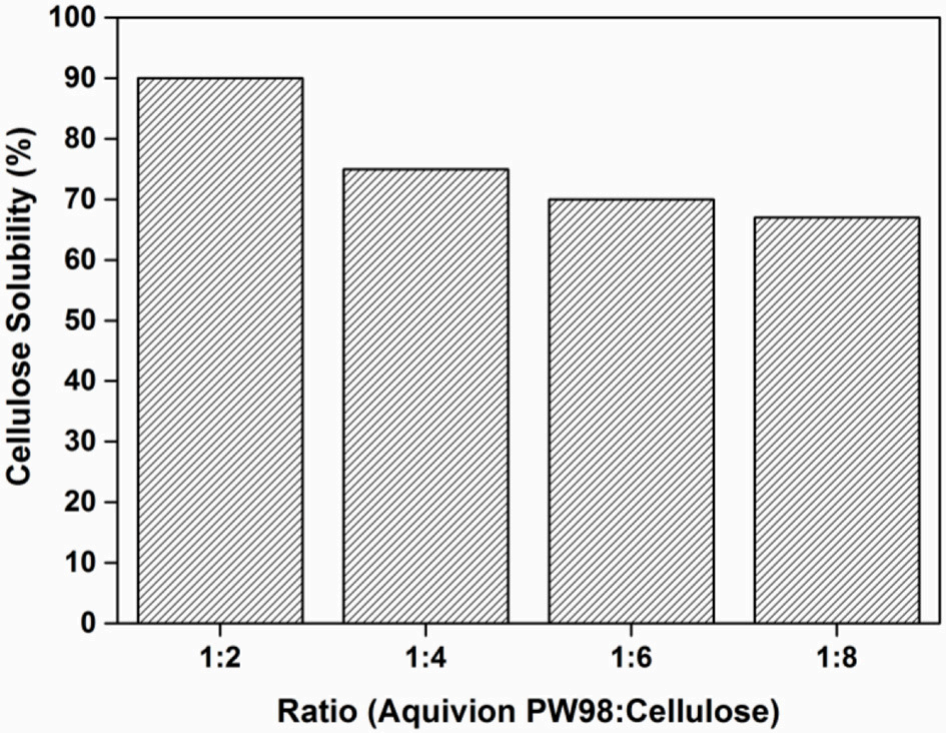
Effect of the Aquivion PW98/cellulose mass ratio on the depolymerization rate of cellulose (mass of cellulose, 1 g; milling speed, 400 rpm; zirconium oxide balls diameter d_MB_, 10 mm; ball-milling time, 24 h).

To support this hypothesis, the amount of cellulose was kept constant (1 g) while varying the amount of Aquivion PW98 (from 500 to 125 mg) during the ball-milling. It corresponds to a variation of the Aquivion PW98/cellulose mass ratio from 1:2 to 1:8 (Figure 2). Interestingly, the water solubility of MCC after the mechanocatalytic reaction did not differ dramatically between both assays, decreasing only from 90 to 70% upon moving from a 1:2 to a 1:8 Aquivion PW98/cellulose mass ratio, after 24 h of ball-milling. Altogether, these results strongly suggest that the depolymerization rate of cellulose under these conditions is not catalytically controlled and that mechanical forces probably play a more important role.

**Figure 3:**
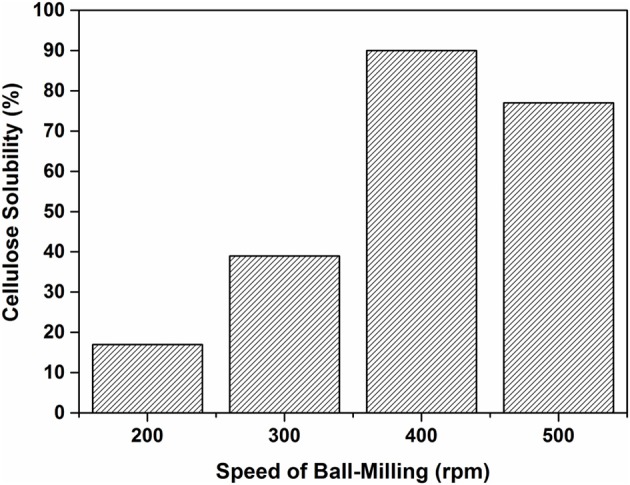
Effect of the bowl stirring speed on the depolymerization rate of cellulose (mass of cellulose, 1 g; Aquivion PW98, 0.5 g; zirconium oxide balls diameter d_MB_, 10 mm; ball-milling time, 24 h).

To further substantiate our assumption, the influence of the ball-milling speed in the process was also studied (Figure 3). During ball-milling, an increase in rotational speed leads to a subsequent rise in apparent energy due to the effective kinetic energy produced as a result of the frequent collisions of the balls between themselves and also between the balls and walls of the reactor. Working at a milling speed of 200 rpm led to a solubility of ~17% after 24 h, which increased by more than 2-folds (38%) when the milling speed was further switched to 300 rpm (Figure 3). At 400 rpm, a striking increase in solubility (90%) was achieved. These results clearly demonstrate that the reaction kinetic is mostly governed by mechanical forces i.e., friction, collisions, shearing, etc, Nonetheless, when the milling speed was further increased to 500 rpm, a reduction of solubility was observed (from 90% at 400 rpm to 77% at 500 rpm). At 500 rpm, colored tar-like insoluble products were formed leading to a decrease in solubility of cellulose after the mechanocatalytic process, due to excessive energy input in this case.

**Figure 4:**
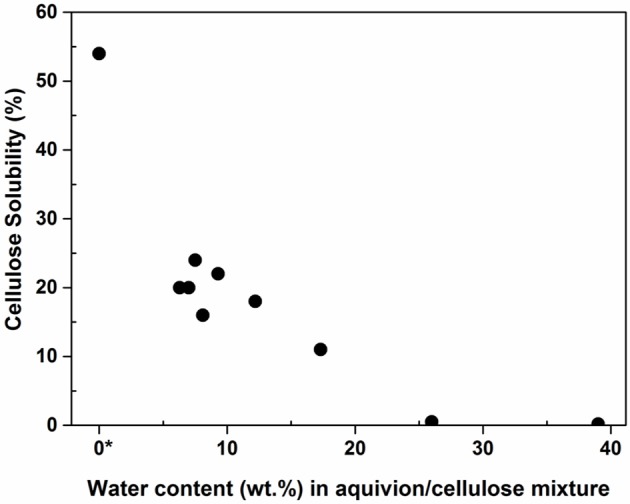
Effect of water on the mechanocatalytic process. The value 0 was arbitrary fixed for the freeze-dried Aquivion PW98/cellulose mixture, although one should note that freeze-dried cellulose still contain chemically adsorbed water, difficult to measure.

Assuming that mechanical forces have a strong impact on the depolymerization rate of cellulose, it occurred to us that the water content of Aquivion PFSA and cellulose will impact the mechanocatalytic process in a significant way, in particular by buffering the mechanical forces. Previously, it has been well-documented that the presence of a liquid, even in trace amount, dramatically impacts a mechanochemical process, a phenomenon known as liquid-assisted grinding (Käldström et al., 2014). Aquivion PW98 and cellulose contained 7 and 6 wt% of water, respectively [i.e., 6.3 wt% of water for the Aquivion PW98/cellulose mixture (0.5:1)]. To assess the role of water, the mixture Aquivion PW98/cellulose was next freeze-dried before the mechanocatalytic process. The reaction was stopped after only 3 h of milling to clearly highlight the role of water. Remarkably, an increase in the mechanocatalytic depolymerization rate was observed and 55% of water soluble products were obtained after only 3 h of ball-milling vs. 20% without freeze-drying. An increase of the mechanocatalytic time from 3 to 6 h led to a nearly complete dissolution of cellulose (90%). i.e., a reduction of the mechanocatalytic treatment time by 4 in comparison to non-freeze dried samples (Figure S1). Then, water was progressively added in order to monitor its effect on the milling. As shown in Figure 4, a sharp drop in the reaction product solubility was observed after 3 h of milling with aliquot amounts of H_2_O, further stressing the important role played by water on the mechanocatalytic process.

**Figure 5:**
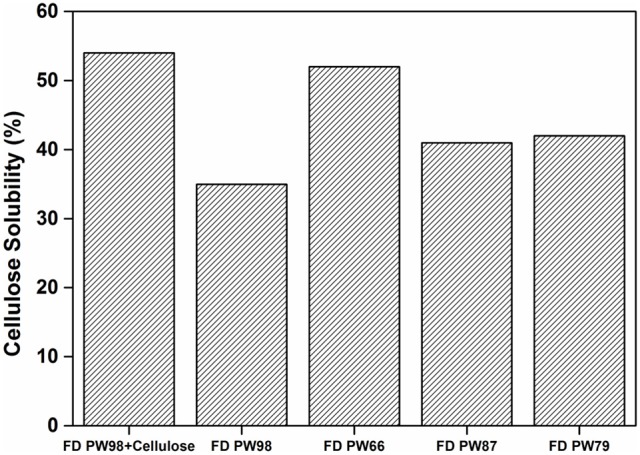
Effect of freeze-drying of Aquivion samples on the mechanocatalytic process. FD, Freeze-dried.

When only Aquivion PW98 was freeze-dried, a slightly lower solubility of cellulose of 40 % was observed (vs. 54% for the freeze-dryied Aquivion PW98/cellulose mixture), indicating that water contained in cellulose was also impacting the mechanocatalytic process to some extent (Figure 5; details of water content estimation is provided in SI, section Materials and Methods). Aquivion PFSA PW98, PW66, PW87 have a similar water content of 7% and thus similarly behaved during the mechanocatalytic depolymerization of cellulose. In contrast, Aquivion PFSA PW79 has a water content of 22 wt% and was significantly less active than other Aquivion samples, providing a cellulose depolymerization product with a water solubility of only 32% after 24 h of ball-milling (Table 1). However, when Aquivion PW79 was freeze-dried before the mechanocataytic process, it behaved similarly than the other Aquivion samples (40% of cellulose was solubilized), further demonstrating the important role played by water (Figure 5). Here again, once freeze-dried, no significant difference of performance, in terms of depolymerization rate of cellulose, was observed between all Aquivion samples, although they have different proton loadings. These results confirm that the kinetics of the mechanocatalytic process is not controlled by the catalyst but mainly by mechanical forces, that can be tuned by the rotational stirring rate or the presence of water.

The water soluble products were analyzed in detail by mass spectrometry (MS), gas chromatography (GC) and high performance anionic-exchange chromatography with pulse amperometric detection (HPAEC-PAD). Consistently with previous reports using H_2_SO_4_ as an acid catalyst, Aquivion catalysts yielded oligosaccharides with a degree of polymerization (DP) up to 11 (MS). Monosaccharides accounted for 13% of the water soluble fraction, formed mainly by free D-glucose (96%) and minor proportions of 1,6-anhydro-D-glucopyranose (levoglucosane, 3%) and 1,5-anhydro-D-glucofuranose (1%) (GC; sample derivatization by sequential oximation-trimethylsilylation reactions as described in the SI). Disaccharides and oligosaccharides with DP 3-11 accounted for 21 and 65% of the water soluble product, respectively (Table 2). No oxidation or degradation product was detected, indicating that the mechanocatalytic process with Aquivion PW98 was fully selective to water soluble oligosaccharides. The disaccharide fraction was analyzed in depth using authentic commercially available standards (GC; sample derivatization by sequential oximation-acetylation reactions as described in the SI). All types of α/β positional regioisomers, namely (1 → 1)-, (1 → 2)-, (1 → 3)-, (1 → 4)-, and (1 → 6)-linked glucobioses, were detected, with the β-(1 → 4) linkage (cellobiose) being dominant (79.5% of the disaccharide fraction). Considering that cellulose exclusively contains β-(1 → 4) glycosidic bonds, these results indicate that cellulose depolymerization during the mechanocatalytic process with Aquivion PW98 proceeds, to some extent, with concomitant self-glycosylation (reversion) reactions, in line with previous reports from Schüth and Beltramini (Meine et al., 2012; Shrotri et al., 2013).

**Table 2:** Relative composition of oligosaccharides linkages recovered after mechanocatalytic depolymerization of cellulose with Aquivion PW98.

**Glycosidic bond (%)**										**Branching pattern (%)**
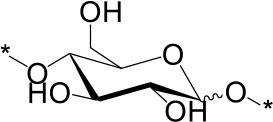		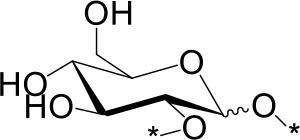		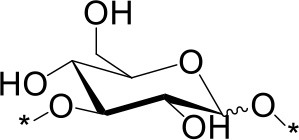		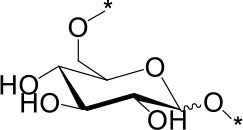		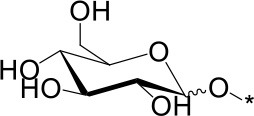		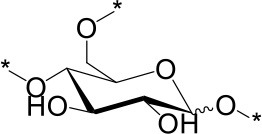
1 → 4		1 → 2		1 → 3		1 → 6		1 → 1′		1 → 4 → 6
79.5		3		3		13		1.5		6
α	β	α	β	α	β	α	β	α,α'	α,β'	–
6.5^a^	73^b^	1.7^c^	1.3^d^	2.3^e^	0.7^f^	6.5^g^	6.6^h^	1^i^	0,5^j^	–

a maltose;

b cellobiose;

c kojibiose;

d laminarabiose;

e nigerose;

f soforose;

g isomaltose;

h gentiobiose;

i trehalose;

j *neotrehalose*.

**Figure 6:**
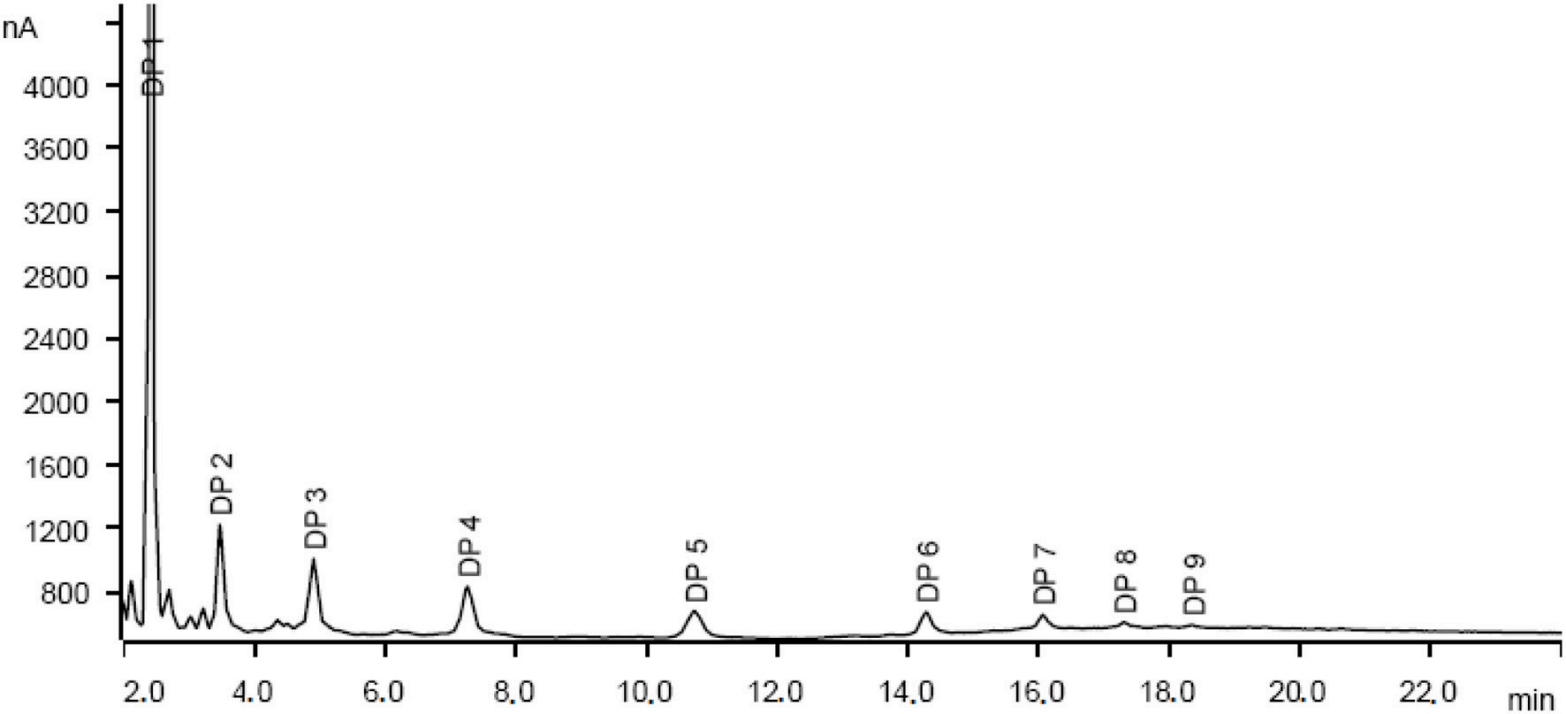
HPAEC-PAC chromatogram (see SI for experimental description) of the oligosaccharide material obtained by the mechanocatalytic depolymerization of cellulose with Aquivion PW 98 (mass of cellulose, 1 g; catalyst, 0.5 g; milling speed, 400 rpm; zirconium oxide balls diameter, d_MB_, 10 mm; ball-milling time, 24 h).

In principle, cross-glycosydation reactions between free glucose and cello-oligosaccharides or between different cello-oligosaccharides could lead to the formation of branched oligosaccharides. To assess the degree of branching, the mixture of oligosaccharides was subjected to a methylation (MeI/NaOH) (Ciucanu and Costello, 2003)—hydrolysis (TFA, 120 °C)-deuteroboration (NaBD_4_)–acetylation (Ac_2_O/TFA) sequence (Kim et al., 2006) prior to GC-MS analysis. This protocol affords the corresponding alditols labeled with deuterium at C-1, methylated at non-glycosylated positions and bearing acetyl groups at positions that were glycosylated in the starting oligosaccharide chain, which can be unequivocally assigned from the corresponding fragmentation patterns in MS by comparison with authentic standards (Sassaki et al., 2005). The data indicated that terminal and monoglycosylated residues accounted for more than 96% of the glucosyl units, the majority of the inner chain glucose units were glycosylated at position O-4 as in cellulose (51.8% of total glucose). Only a small proportion (3.8%) of the inner residues are doubly glycosylated at positions O-4 and O-6, supporting that most of the oligosaccharide material keep the (1 → 4)-glycosylation pattern of the parent polysaccharide. Cellulose depolymerization is therefore the predominant reaction occurring under these conditions. Indeed, the HPAEC.PAD chromatogram obtained from the crude reaction material showed a profile compatible with the major presence of cello-oligosaccharides of increasing DP. Quantitative analysis indicated that over 93% of the oligosaccharide material is comprised in the DP 2-to-6 fraction (Figure 6).

Noteworthy, the as-obtained oligosaccharides were highly hygroscopic. Water playing an important role in the reaction, the mechanocatalytic process was additionally performed under an argon atmosphere to assess a possible negative effect of water coming from the hygroscopicity of oligosaccharides during the milling. No significant change was observed as compared with the mechanocataytic process conducted under air, however, suggesting that mainly water initially contained in cellulose and Aquivion was responsible for the buffering of mechanical forces. This is an important point as regards industrial perspectives. Indeed, working in air will reduce the complexity of the system leading a more robust and lean process easier to be performed.

## Conclusions

We have demonstrated that Aquivion, a sulfonated perfluorinated ionomer, was capable of promoting the selective depolymerization of cellulose to afford water-soluble oligosaccharides with a DP up to 11. The detailed characterization of the water soluble fraction revealed that depolymerization and reversion reactions concomitantly occurred during the mechanocatalytic process, although the first largely predominated. The kinetic of the reaction is governed by mechanical forces, i.e., friction, collisions, shearing, etc. More importantly, we discovered that water contained in Aquivion and cellulose lowered the efficiency of the mechanocatalytic process, presumably by buffering mechanical forces. The plasticization effect of water in Aquivion is known although never reported in this frame. A removal of water before the mechanocatalytic process has permitted reducing the milling time. Under optimized conditions, yields of water soluble sugars of up to 90 and 97% were achieved using Aquivion PW98 and PW66, respectively. In comparison to H_2_SO_4_ which is commonly used in such application, Aquivion is easily separated from soluble sugars (dissolution of sugars in water and filtration of Aquivion), thus opening interesting perspectives to investigate the physicochemical properties of the as-obtained oligosaccharides or to further chemically process these sugars into specialty chemicals.

## Author contributions

AK and PA performed the mechanocatalytic depolymerization of cellulose. KD and JG were in charge of the characterization of water soluble products obtained after the mechanocatalytic process. BE and SM were in charge of the critical role of water. CO was in charge of Aquivion catalysts (preparation, proton loading, water content, etc.). FJ supervized the work.

### Conflict of interest statement

The authors declare that the research was conducted in the absence of any commercial or financial relationships that could be construed as a potential conflict of interest.
