# Supplementary material for: Mechanocatalytic Depolymerization of Cellulose With Perfluorinated Sulfonic Acid Ionomers
Source: Front Chem. 2018 Mar 22;6:74. doi: 10.3389/fchem.2018.00074 (PMC5874291; doi:10.3389/fchem.2018.00074)
Supplement: Supplementary file 1 [file Presentation1.PDF]

Supporting Information

Mechanocatalytic depolymerization of cellulose with perfluorinated sulfonic acid ionomers

Ayman Karam,[1,2#] Prince N. Amaniampong, [1#] José M. García Fernández,[3] Claudio Oldani,[4] Karine Vigier and François Jérôme, [1,2*]

[1] INCREASE (FR CNRS 3707), ENSIP, 1 rue Marcel Doré, TSA 41105, 86073 Poitiers (France)
[2] Institut de Chimie des Milieux et Matériaux de Poitiers (IC2MP), Université de Poitiers, CNRS, ENSIP, 1 rue Marcel Doré, TSA 41105, 86073 Poitiers (France).
[3] Instituto de Investigaciones Químicas (IIQ), CSIC - University of Sevilla, Avda. Americo Vespucio 49, E-41092 Sevilla, Spain
[4] Solvay Speciality Polymers, Viale Lombardia 20, 20021 Bollate MI, Italy

Corresponding author: francois.jerome@univ-poitiers.fr
# Authors contributed equally to the manuscript

1. Catalyst synthesis and mechanocatalytic depolymerization of cellulose

Catalysis synthesis

SBA-15-SO_3H catalyst was prepared following a reported procedure (Karam *et al.*, 2007). In a typical synthesis process, pluronic (4g) was dissolved in 125g of aqueous HCl (1.9M) and stirred at room temperature. The solution was then heated at 40°C before addition of 7.7 g (0.0369 mol) of TEOS. After stirring for 45mn, MPTMS (0.8 g, 0.0041 mol) and 0.0369 mol of 35% H_2O_2 was added. The solution was then stirred for 24h at 40°C and aged into a teflon autoclave for an additional 24h at 100°C. The resulting solid was finally collected by filtration and thoroughly washed with water. The recovered SBA-15-SO_3H was dried in an oven at 50°C for 18h.

CMK-3-SO_3H was synthesized *via* a reported procedure by Jun et al.,(Jun *et al.*, 2000). Typically, the calcined SBA-15 was impregnated with aqueous solution of sucrose containing sulfuric acid, 1 g of SBA-15 was added to a solution obtained by dissolving 1.25 g of sucrose and 0.14 g of H_2SO_4 in 5g of H_2O. The mixture was placed in a drying oven for 6h at 373K, and subsequently the oven temperature was increased to 433 K and maintained there for 6 h. The sample turned dark brown or black during the treatment in the oven. The silica sample, containing partially polymerized and carbonized sucrose at the present step, was treated again at 373 and 433 K using the same drying oven after the addition of 0.8 g of sucrose, 0.09 g of H_2SO_4 and 5g of H_2O. The carbonization was completed by pyrolysis with heating to typically 1173 K under vacuum. The carbon - silica composite obtained after pyrolysis was washed with 1 M NaOH solution (50 vol % ethanol - 50 vol % H_2O) twice at 373 K or 5 wt % hydrofluoric acid at room temperature, to remove the silica template. The template-free carbon product thus obtained was

filtered, washed with ethanol, and dried at 393 K. Thereafter, the recovered mesoporous carbon (so-called CMK-3) was suspended in concentrated H_2SO_4 (1g of solid per 20mL of acid) and stirred overnight. The CMK-3-SO_3H was washed several times with distilled water and then dried in an oven at 60°C overnight.

Aquivion PW66, PW79, PW87 and PW98 were used without further pretreatment as received from Solvay Specialty Polymers.

Mechanocatalytic depolymerization of cellulose

Various amounts of cellulose and catalyst were ground using a planetary ball-mill (Retsch MP100). The mixture of catalyst and cellulose were ground in a 125 mL bowl made of Zirconium Oxide, utilizing 20 of 10 mm balls made of the same material as the milling bowl. The experiments were performed at desired rate for a desired time as described in the main manuscript for each conditions investigated.

Determination of solubility

After each milling, the milled mixture of cellulose and catalyst was recovered. The determination of solubility involved three parts, dispersion, filtration and drying. The dispersion is carried out in a 20 mL flacon, weighing 300 mg of the solid mixture and 20 mL distilled water, stirring and leaving it in an ultrasonic bath for 2 hours. The mixture is filtered through a 47 mm Millipore Pyrex Filter Holder; the PFTE filter has a pore size of 0.22 µm. The filter containing the solid after filtration is placed in a petri dish in the oven at 60 °C overnight. The final mass is measured by the difference between the filter containing the dry solid at ambient temperature and the initial mass of the filter.

2. Estimation of water content in cellulose and Aquivion catalysts

5 g of cellulose was freeze-dried overnight, the freeze-dried sample was weighed thereafter and the loss of weight recorded as the amount of water. The same procedure was repeated for the Aquivion catalysts.

3. Effect of Aquivion/Cellulose Freeze-drying

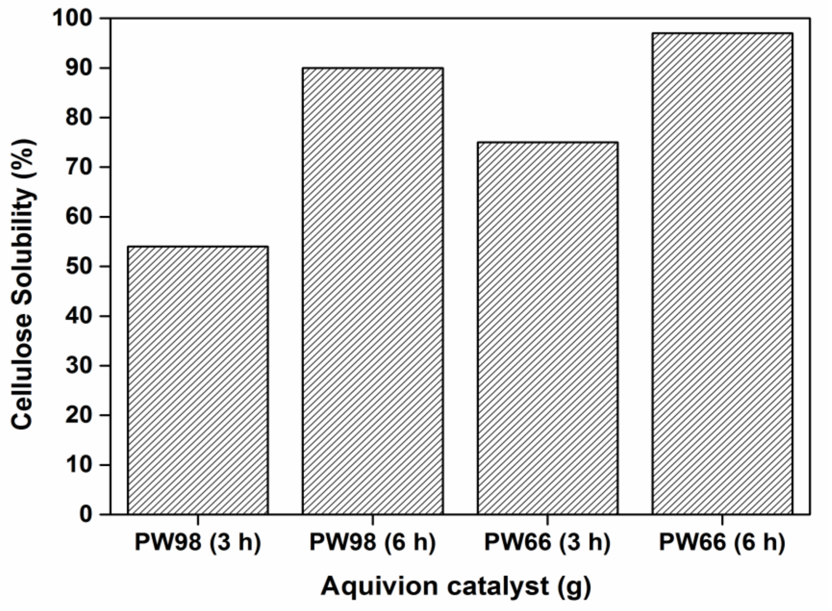

Figure S1. Reaction conditions: Mass of cellulose, 1 (g); catalyst, 0.5 (g), Zirconium Oxide balls diameter d_{MB}, 10 mm.

4. GC analysis of bond linkages of produced water soluble oligosaccharides

GC-FID was carried out using an Agilent 6890 Series Plus chromatograph with an EPC injector fitted with a cross-linked 5% phenyl-dimethylsiloxane column (HP-5; 30 m x 320 µm x 0.25 µm). Operating conditions were: injection port temperature 310 ºC; splitting ratio 25:1; injection volume 1 µL of derivatized samples; column oven temperature programmed from 180 to 310 ºC at 5 ºC min^{-1}, with a 5 min hold at 310 ºC; carrier gas helium (constant flow at 1.2 mL min^{-1}); detector port temperature 325 ºC. Total acquisition time was 31 min.

For GC-FID analysis of the monosaccharide fraction, the sugar components were first transformed into their corresponding per-*O*-trimethylsilyl (TMS; nonreducing sugars) or per-*O*-trimethylsilylated oxime (TMS-oximes; reducing sugars) derivatives. The crude samples were diluted with deionized water (1 mL) and the aqueous solutions were freezedried. To 15-20 mg of each sample, deionized water (1 mL) was added. To 100 µL of the resulting solution was then added 100 µL of internal standard (I.S.; 4 mg mL^{-1} phenyl β-D-glucopyranoside in acetone-water 1:9, v/v) and the final solution was evaporated to dryness at 60 ºC (drying oven). The residue was treated with 1 mL of a solution of hydroxylamine in pyridine (20 mg mL^{-1}) at 60 ºC over 50 min, with mixing at intervals. Neat hexamethyldisilazane (200 µL) and trimethylchlorosilane (100 µL) were then added, and the reaction mixtures were kept at 60 ºC over a further 40 min period. Formation of a white precipitate was observed during this operation, which was separated by centrifugation (13 000 rpm, 5 min) before injection in the GC apparatus. It is worth noting that following oximation-trimethylsilylation derivatization, reducing compounds (e.g., residual D-glucose) provide two peaks in the GC chromatogram,

corresponding to the *syn*- and *anti*-TMS-oximes, while nonreducing derivatives (e.g., levoglucosane and the I.S.) provide a single peak.

For GC-FID analysis of the glucodisaccharide fraction, the sugar components were instead transformed into the corresponding aldononitrile peracetates. To 100 μL of a 16 mg mL^{-1} solution of the polymannoside sample in water, 100 μL of a solution of phenyl β-D-glucopyranoside (internal standard) in acetone-water (1:9 v/v, 4 mg mL^{-1}) was added and lyophilized. Derivatization was next conducted according to the procedure of Zhang and Amelung (Zhang *et al.*, 2007). The derivation reagent (0.3 mL), which contained 32 mg.mL^{-1} hydroxylamine hydrochloride and 40 mg.mL^{-1} of 4-(*N*,*N*-dimethylamino)pyridine (DMAP) in pyridine–methanol (4:1, v/v), was added to a vial containing the dry sample and the internal standard. The capped vial was shaken and heated for 50 min at 60 ºC. Then, the vial was cooled to room temperature and 1 mL of Ac_2O was added. The vial was closed, shaken again, kept at room temperature for 40 min and concentrated under vacuum at 40 ºC. The residue was dissolved in 0.1 mL CH_2Cl_2 and centrifuged (8.5 rpm, 2.5 min) before injection in the GC apparatus. The identity of mannodisaccharides in the samples was confirmed by comparison of the GC chromatograms with that of authentic samples.

5. Determination of the branching pattern of the samples obtained by mechanolytic depolymerization of cellulose by GC-MS analysis of the methylation-hydrolysis-reduction-acetylation products.

GC-MS was carried out using a Shimadzu GC-2010 chromatograph fitted with a cross-linked 5% phenyl-dimethylsiloxane column (ZB-5MS; 10 m × 0,18 mm × 0,18 mm) and connected to a Shimadzu GCMS-QP2010 Plus mass spectrometer. The ionization potential was 70 eV, and spectra were recorded in low-resolution mode. Operating conditions were: injection port temperature 275 ºC; splitting ratio 5:1; injection volume 1 μL of derivatized samples; the temperature programme was set as follows: the initial column temperature of 120 ºC was held for 1 min and then the temperature was increased at 20 ºC min^{-1} to 250 ºC, with a 2.67 min hold at 250 ºC; carrier gas helium (constant flow at 0.7 mL min^{-1}). Total acquisition time was 30 min.

For derivatization, vacuum-desiccated samples were methylated by using the method of Ciucanu and Costello (Ciucanu & Costello, 2003). Typically, the sample (1 mg) was dissolved in 10 μL of water and 0.5 mL of DMSO was added under stirring. Methyl iodide (50 μL) and finely powdered sodium hydroxide (5 mg) were added to the solution and stirred vigorously for 1 min to get a suspension. After this, a larger amount of sodium hydroxide (15 mg) was added to the contents of the vial and the mixture was stirred at room temperature for 10 min. The samples were partitioned with 2 mL of water and 2 mL of CH_2Cl_2 and the organic phase was washed with 3-4 mL of water and dried under a stream of nitrogen. To ensure complete methylation, this process was repeated a next time. Further hydrolysis, reduction with $NaBD_4$ and subsequent acetylation were performed according to Kim et al (Kim *et al.*, 2006). Briefly, the *per*methylated product was hydrolyzed into the monosaccharide constituents by treatment with 2 M TFA (250 μL) at 120 ºC for 2 h. TFA was then removed under vacuum by co-evaporation with drops of methanol. The dry residue was dissolved in aqueous $NaBD_4$ (10 mg

mL^{-1}, 100 μL) and the solution was magnetically stirred at room temperature for 1 h. The reaction was then stopped by addition of a drop of glacial AcOH and dried by co-evaporation with methanol (3 × 100 μL). The mixture of partially methylated alditols thus obtained was acetylated by reaction with Ac_2O (100 μL) and TFA (80 μL) at 50 ºC for 10 min, then dried by co-evaporation with acetone, partitioned between CH_2Cl_2 (2 mL) and water (4 × 2 mL), the organic phase was dried (Na_2SO_4) and concentrated to dryness. Finally, the residue was re-dissolved in CH_2Cl_2 (50 μL) before injection in the GC-MS. Sugar identification was based on their retention times and mass spectra compared to those of authentic standards (Sassaki *et al.*, 2005).

6. Analysis of cellooligosaccharides by HPAEC-PAD

Samples (1 mg mL^{-1} in water) were analyzed on a Metrohm 850 Professional IC system with a 919 IC auto-sampler plus, dual pumps and a PAD (Herisau, Switzerland). Each aliquot of 20 μL was injected on a CarboPac PA100 guard (4 × 50 mm, Dionex, Sunnyvale, CA) connected to an analytical column (4 × 250 mm, Dionex, Sunnyvale, CA) at a flow rate of 1 mL/min. The analysis was carried out at 30 ºC with a linear gradient, in which the eluent A was 100 mM NaOH, and eluent B was 450 mM NaOAc in 100 mM NaOH. A linear gradient from 0% to 60% of mobile phase B was eluted for 35 min. The PAD data was acquired and analyzed with software MagIC Net 2.4 (Herisau, Switzerland). The identity of D-glucose and cello-oligosaccharides from DP 2 to 7 was confirmed by comparison with authentic standards.

References

Ciucanu, I. & Costello, C. E. (2003) Elimination of oxidative degradation during the per-O-methylation of carbohydrates. *Journal of the American Chemical Society,* **125**, 16213-16219.

Jun, S., Joo, S. H., Ryoo, R., Kruk, M., Jaroniec, M., Liu, Z., *et al.* (2000) Synthesis of new, nanoporous carbon with hexagonally ordered mesostructure. *Journal of the American Chemical Society,* **122**, 10712-10713.

Karam, A., Gu, Y., Jérôme, F., Douliez, J.-P. & Barrault, J. (2007) Significant enhancement on selectivity in silica supported sulfonic acids catalyzed reactions. *Chemical Communications,* 2222-2224.

Kim, J. S., Reuhs, B. L., Michon, F., Kaiser, R. E. & Arumugham, R. G. (2006) Addition of glycerol for improved methylation linkage analysis of polysaccharides. *Carbohydrate research*, **341**, 1061-1064.

Sassaki, G. L., Gorin, P. A., Souza, L. M., Czelusniak, P. A. & Iacomini, M. (2005) Rapid synthesis of partially O-methylated alditol acetate standards for GC–MS: Some relative activities of hydroxyl groups of methyl glycopyranosides on Purdie methylation. *Carbohydrate Research*, **340**, 731-739.

Zhang, W., He, H. & Zhang, X. (2007) Determination of neutral sugars in soil by capillary gas chromatography after derivatization to aldononitrile acetates. *Soil Biology and Biochemistry*, **39**, 2665-2669.
